# Non-Communicable Diseases-Related Stigma: A Mixed-Methods Systematic Review

**DOI:** 10.3390/ijerph17186657

**Published:** 2020-09-12

**Authors:** Sarju Sing Rai, Elena V. Syurina, Ruth M. H. Peters, Annisa Ika Putri, Marjolein B. M. Zweekhorst

**Affiliations:** 1 Athena Institute, Faculty of Science, Vrije University Amsterdam, 1081 Amsterdam, The Netherlands; e.v.syurina@vu.nl (E.V.S.); r.m.h.peters@vu.nl (R.M.H.P.); annisa.ika@gmail.com (A.I.P.); m.b.m.zweekhorst@vu.nl (M.B.M.Z.); 2 Barcelona Institute for Global Health (ISGlobal), University of Barcelona, 08007 Barcelona, Spain

**Keywords:** stigma, discrimination, non-communicable disease, NCD, systematic review

## Abstract

This systematic review examines and consolidates existing evidence on stigma associated with the top four non-communicable diseases (NCDs)—cancers, cardiovascular diseases, chronic respiratory diseases, and diabetes—and its impact on the lives of people affected. We conducted a systematic literature search in PubMed, PsycINFO, JSTOR, Science Direct, and Web of Science for original research in English that explored health-related stigma among people living with either of the four NCDs. A three-step integrative synthesis of data was conducted. Twenty-six articles (qualitative = 15; quantitative = 11) were selected, with most (*n* = 15) related to cancers, followed by diabetes (*n* = 7), chronic respiratory diseases (*n* = 3), and cardiovascular diseases (*n* = 1). Blame, shame, and fear were the main causes of stigma, the origin and nature of which differed according to the disease-specific features. The manifestations (enacted and felt stigma) and consequences (social, behavioral, psychological, and medical) of stigma across NCDs were similar. Inconsistencies existed in the conceptualization of stigma processes. To fill this gap, we developed an NCD-related stigma framework. People living with NCDs can experience stigma, which can negatively impact their health, management of their disease, and quality of life. The new framework can help in improving the understanding of the processes and experiences of stigma related to NCDs.

## 1. Introduction

Non-communicable diseases (NCDs) are the leading cause of death in the world [1]. Over the last two decades, NCDs have been on the rise globally, and now account for more than one-half of the global burden of disease [1,2]. WHO (World Health Organization, Geneva, Switzerland) estimates that the four main NCDs—cardiovascular diseases (CVDs), cancers, diabetes, and chronic respiratory diseases (CRDs)—account for 70% of the 56.4 million deaths that happened globally in 2015 [1]. Besides mortality, NCDs are also responsible for high rates of morbidity and disability, resulting in low productivity, catastrophic costs of care, and lower psychosocial well-being and quality of life of persons affected [3,4,5]. Low- and middle-income countries (LMICs) in particular are disproportionately burdened by NCDs with occurrence of 86% of premature deaths [6]. This is estimated to result in cumulative economic losses of USD 7 trillion over the next decade causing a surge in poverty in the LMICs [6].

Stigma related to infectious diseases like HIV/AIDS and leprosy, and mental illnesses like schizophrenia has garnered global attention because of its resounding negative impact on the people affected, the treatment and management of their disease, and ultimately the effectiveness of public health programs globally [7]. Emerging evidence suggests that people living with NCDs, like those with infectious diseases, may also experience stigma and discrimination because of their health condition [8,9,10,11,12]. Considering the growing burden of NCDs globally, it is imperative to have a better understanding of stigma related to NCDs.

Goffman first described stigma in his seminal work as an attribute that is discredited by society, leading to a “spoilt identity” of the persons affected [13]. The conceptualization of stigma has since evolved over the years, with improved understanding of the mechanisms and processes of stigmatization. One of such advanced concepts was provided by Link and Phelan, where they described stigma as a result of “labelling, stereotyping, status loss, and discrimination” facilitated by the power dynamics that exist in society [14]. The conceptualization of stigma has also been adapted in health research to address the stigmatization faced by people living with/affected by different health conditions. Weiss et al. defined such health-related stigma as “a social process, characterized by ‘exclusion, rejection, blame or devaluation’ resulting from experience, perception, or anticipation of negative social judgement owing to a person’s health condition” [15]. Many theories and frameworks on health-related stigma have now been developed for different disciplines (public health, sociology, psychology, psychiatry) and also different health conditions (e.g., infectious diseases like HIV and leprosy, and mental illnesses like schizophrenia) [14,15,16,17,18,19,20,21,22]. However, a commonality that exists in these varied conceptualizations is the differentiation of stigma into that concerning those who stigmatize (public or social stigma), and those who are stigmatized (personal or individual stigma) [15,17,23]. Public/social stigma represents the negative perception and judgement that exist in society and among societal actors that are known to perpetuate and facilitate the stigmatization of individuals with health conditions, whereas personal/individual stigma deals with the experiences and perception of stigma from the perspective of those with/affected by a certain health condition(s) [16,24]. Stigma at the personal/individual level is considered more detrimental as it is known to discourage people affected from disclosing their health condition, accessing healthcare services, and adhering to a treatment regimen [20,23,25,26]. Scambler et al. further studied the phenomenon of stigmatization among people living with health conditions, and reported on the two types of personal stigma manifestations they deal with: (1) enacted stigma—also called discrimination or experienced stigma, which is an actual experience of stigmatizing acts, attitudes, and behavior from others because of having the stigmatized condition; and (2) felt stigma—which is often built upon perceived social exclusionary views towards those with stigmatized health conditions, and is characterized by feelings of shame for having the disease and the fear of encountering enacted stigma because of the diseased status [20].

Many people living with cancers, CVDs, CRDs, and diabetes are known to encounter similar stigma-related manifestations like avoidance and isolation [8,9,27], exclusion from social participation [28,29,30], both perceived and experienced unfair and unjust treatment in social, education, employment, and healthcare settings [8,9,31,32,33], feelings of shame and guilt for having the disease [11,27,31,34], and fear of discrimination and ostracization [32,35,36]. Such experiences are known to discourage help-seeking and the disclosure of health status, which in turn can potentially hamper the treatment and management of these conditions [8,33,37,38].

However, unlike stigma associated with infectious diseases, NCD-related stigma is yet to receive enough recognition within the field of public health. This is reflected in the existing policies, research, and care practices related to NCDs. Even though numerous policies, strategies, and plans of action have been developed to tackle NCDs, stigma has been widely absent in the strategy formulation process [1,15]. The UN Political Declaration on NCDs [39] and the WHO Global Action Plan for the Prevention and Control of NCDs (2013–2020) [6] have completely left out stigma from their scope of work, while the NCD Alliance Strategic Plan (2016–2020) does mention stigma but fails to specify any substantial action plan to address it [40]. In the context of research, a number of studies have been carried out on stigma associated with cancers, but these are greatly fragmented and inconsistent in regard to the conceptualization and exploration of stigma [41,42]. Only few studies have explored stigma associated with the other prevalent non-communicable conditions, such as CVDs, CRDs, and diabetes [9,12,28,34]. Further, there are some reviews that have specifically focused on stigma related to specific NCDs like sickle cell disease [43], dementia [44] and other neurological diseases [45], respiratory diseases [42], and mental illnesses [46,47]. However, a comprehensive and wide-reaching understanding of NCD-related stigma is still needed to develop effective strategies to best respond to the growing epidemic of NCDs. So far, no single research has systematically examined the differences and similarities among the features and experiences of stigma across the four main NCDs, their impact on the lives of persons affected, and the wider implications on the management and control of NCDs. This review will focus on the four most prevalent NCDs, which are cancers, CVDs, CRDs, and diabetes, because they are considered the four main NCDs that are the leading causes of death globally. WHO has specifically identified these four conditions of utmost importance and developed a seven-year Global Action Plan (2013–2020) for their prevention and control [6]. This review aims to examine the current evidence on stigma and stigma-related experiences and their causative factors, consequences, and mitigating factors across the four main NCDs—cancers, CVDs, CRDs, and diabetes.

## 2. Methods

We developed and followed a standard systematic review protocol (systematic review registration-PROSPERO 2018: CRD42018089218) in accordance with the PRISMA statement and checklist (see Supplementary Table S1) [48]. Two reviewers (SSR and AIP) conducted the search and extracted data.

### 2.1. Search Strategy

We conducted a systematic literature search in five databases (PubMed, Web of Science, JSTOR, PsycINFO, and Science Direct) indexed up to February 2018. Search syntaxes were constructed iteratively using keywords related to stigma, the four NCDs, and their relevant synonyms. The search terms and syntaxes are shown in Supplementary Table S2.

### 2.2. Article Search

The initial search yielded 1026 studies (database search = 1011 studies; hand search = 15 studies). After removal of 299 duplicates, the titles and abstracts of the remaining 727 studies were screened using the following eligibility criteria.

#### 2.2.1. Inclusion Criteria

1. Peer-reviewed articles in English;
2. Studies on stigmatized persons living with one of the following four NCDs: cancers, CVDs, CRDs, and diabetes;
3. Health-related stigma being the main focus of the study (qualitative) or the dependent variable (quantitative).

#### 2.2.2. Exclusion Criteria

1. Not original research (e.g., review, editorial, opinion pieces, commentary, protocol, etc.)

**Figure 1:**
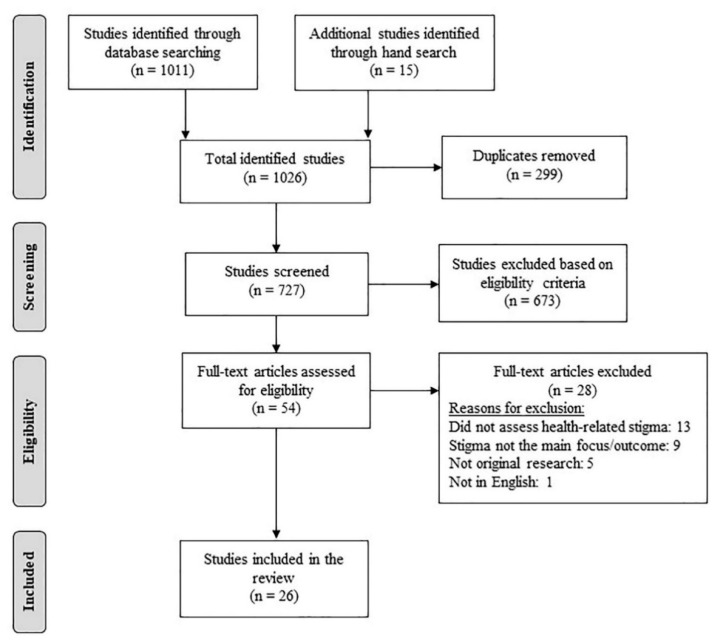
PRISMA flowchart of study selection process.

After the full-text screening, 54 articles were assessed for eligibility, following which 26 articles were finally selected for the review. Figure 1 illustrates the study selection process conducted as per the PRISMA guidelines.

### 2.3. Data Extraction

Two reviewers (SSR and AIP) independently extracted the data into a standardized data extraction matrix designed and piloted by a third reviewer (EVS). The extracted data were cross-checked and compared by the two reviewers (SSR and AIP) and any discrepancies were discussed and resolved. The following information was retrieved during data extraction: study location, design, participants, type of stigma and NCD studied, measurement tools and their quality, prevalence and/or level of stigma, and related factors/predictors/correlates (sources of stigma, experiences of stigma, causative factors of stigma, its reported consequences, and mitigating factors/strategies).

### 2.4. Data Synthesis

Synthesis of the quantitative and qualitative data was carried out adapting the Evidence for Policy and Practice Information and Coordinating Centre (EPPI-Centre) method for integrative synthesis of qualitative and quantitative research for systematic reviews [49]. As per the method, data were synthesized in three steps: (i) quantitative synthesis, (ii) qualitative synthesis, and (iii) final integrative synthesis.

#### 2.4.1. Quantitative Synthesis

Due to the heterogeneity in regard to the tools used and variables explored, a meta-analysis of the quantitative data was not possible. Hence, the findings were descriptively compiled and reported. Data on prevalence (expressed in percentage of participants who reported stigma) and/or levels of measured stigma (mean, SD), and the associations with predictors/correlates of stigma (effect sizes) from quantitative studies were descriptively presented. Measures of group differences (e.g., differences in mean, odds ratio, risk ratio) or associations (e.g., *B, β, r*) were compiled to illustrate the effect sizes of the explored associations. In case the effect sizes were unavailable, Cohen’s *d* was calculated where possible [50]. As all quantitative studies were cross-sectional in design, no causal and temporal relationships were inferred.

#### 2.4.2. Qualitative Synthesis

The qualitative data were thematically analyzed and synthesized [49]. The findings from qualitative studies (relevant texts, quotes, and tables) and constructs/concepts detailed in the quantitative studies (found in the introduction, framework, stigma scale/tools, and results) were evaluated (based on the content, relevance, and relationship to the categories), then thematically classified into five categories—facilitators, drivers, experiences, consequences, and mitigation of stigma. The thematic categories were compared to an extant theoretical framework developed by Schabert et al. [51] for the conceptualization of diabetes-related stigma. The framework was chosen for its comprehensiveness and broader scope, which includes the sources, mechanisms, experiences, consequences, and mitigating factors of stigma. However, during piloting, we discovered the framework could not well address the variations that existed across the four different conditions as it specifically catered only to type 2 diabetes. We then revised the framework in order to address and accommodate the different features and experiences of stigma related to the four NCDs. The following revisions were incorporated into the framework:

1. We used Pryor and Reeder’s [2] conceptual model of stigma to categorize the sources of stigma into two groups—structural and societal actors (public).
2. We expanded the psychosocial mechanisms of stigma from Schabert et al.’s [51] framework to Engel’s [52] biopsychosocial model which could better group and explain the mechanisms that drive stigma across different NCDs.
3. We re-categorized the experiences/manifestations of stigma as per Scambler’s hidden distress model of stigma [20,26] into enacted stigma and felt stigma. We further extended the concept of felt stigma as per Weiss [53] and Tabah [54] into perceived stigma (perceptions about how societal actors negatively view and judge those with the health condition), internalized stigma (acceptance of stigmatizing social views and resultant feeling of shame for having the disease), and anticipated stigma (fear of encountering enacted stigma because of one’s health condition). In this review, perceived stigma and anticipated stigma are categorized as two distinct manifestations of felt stigma, the former arising from the awareness of stigma in society and the perception of stigmatization as a result of it, and the latter from anticipation and fear of discrimination [21,55]. Further, as per the conceptualization of stigma by Moore [55] and Weiss [53], in this study, internalized stigma is not confounded as self-stigma but as one of its components related to endorsement/acceptance of stereotypes and feelings of devaluation as a result of it. In this review, we will not specifically discuss self-stigma as a separate category, but reference it as a broader construct as described by Corrigan [17] that encompasses concepts of awareness of social norms and stereotypes, acceptance, endorsement, and application of those in one’s life, and the resultant consequences.
4. Further, we expanded the consequences of stigma to include social, psychological, behavioral, and medical consequences.
5. We also categorized the mitigating strategies into two distinct groups—coping strategies (internal/personal) and support/resources (external).

**Figure 2:**
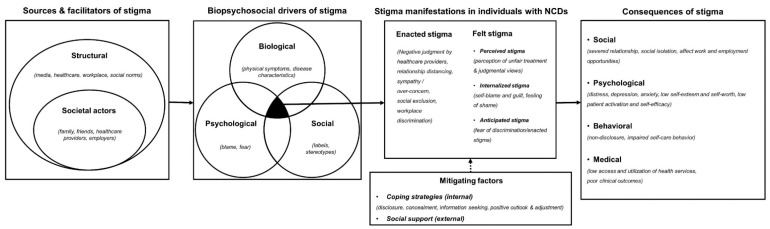
A revised framework for understanding non-communicable disease (NCD)-related stigma.

The revised framework provides improved conceptualization and classification of the study results into five main categories: (1) sources of stigma—that perpetuate stigmatization of people with NCDs, (2) biopsychosocial mechanisms/drivers—that explain the causative mechanism of stigmatization, (3) manifestations of stigma in people living with NCDs, (4) consequences of stigma, and (5) mitigating factors/strategies to curb/overcome stigma. Figure 2 shows the new revised model for NCD-related stigma.

#### 2.4.3. Integrative Synthesis

A matrix was developed integrating both the quantitative and qualitative data into the theoretical framework to descriptively summarize the findings (presented in Supplementary Table S4). The final results are narratively reported in the Results section assimilating both the thematic findings obtained from qualitative studies, and relevant data (frequencies and statistical associations with effect sizes) from quantitative studies. The quantitative studies included in this review mostly examined the associations between stigma and its different consequences (social, psychological, behavioral, and medical) using inferential statistics. Hence, the four thematic categories (sources, biopsychosocial drivers, manifestations, and mitigating factors) were narratively reported integrating both qualitative (examples, quotes, excerpts) and quantitative findings (descriptive data—frequency and percentages), and where available, inferential data (effect sizes). For the fifth category (consequences), both qualitative findings and inferential data with effect sizes were reported.

The overall data analyses and synthesis were conducted independently by two reviewers (SSR and AIP), and the results were finalized after discussion and consensus among the reviewers (SSR, AIP, and EVS). Discussion and consensus among reviewers (SSR, AIP, and EVS) were maintained in every step of the analytical process to ensure sufficient rigor and robustness.

### 2.5. Quality Assessment

The methodological quality of all included studies was assessed independently by the two reviewers (SSR and AIP) using 14-item criteria for quantitative, and 10-item criteria for qualitative studies developed by Kmet et al. [56]. The articles were scored, and for each criterion a score between zero and two was assigned. The total score was calculated and converted into an index score by dividing the total calculated score by the full attainable score. An index score of 0.59 and below was considered unsatisfactory, between 0.60 and 0.79 was considered satisfactory, and 0.80 and over was considered very good [56,57]. The index scores assigned by both independent reviewers (SSR and AIP) were compared. In case of a difference less than 0.2, an average score was calculated and assigned as the difference was not considered significant. When the difference between the scores was equal to or greater than 0.2, the discrepancies were discussed until consensus was reached. The quality assessment matrix is presented in Supplementary Table S3.

## 3. Results

A total of 26 articles (15 qualitative and 11 quantitative) were included. Most studies (*n* = 15) were on stigma related to cancers (miscellaneous cancers = 4; lung = 4; breast = 3; cervical = 2; prostate = 1; head and neck = 1), followed by diabetes (*n* = 7; Type1 = 2; Type2 = 5), CRDs (*n* = 3; COPD = 2; asthma = 1), and CVDs (*n* = 1; stroke-related stigma). Except for one article on stroke, no other studies on CVDs were identified. Most of the studies were conducted in high-income countries (*n* = 21) with seven studies from the USA, followed by Australia (*n* = 3), Japan (*n* = 3), Korea (*n* = 2), the UK (*n* = 2), and one each from Norway, Switzerland, and Singapore. Out of the five LMICs, two were from India and one each from Ghana, Uganda, and Thailand. All studies had a cross-sectional design.

### 3.1. Types of Stigma and Definitions

Most studies were on felt stigma (perceived = 11; internalized = 8; anticipated = 4), followed by enacted stigma (*n* = 9). Four studies—which were all qualitative—did not specify the type of stigma studied and only discussed the overarching generalized concept of stigma [33,34,35,36]. Table 1 shows an overview of the different types of stigma explored in the selected studies.

The studies also used varied definitions of the type and nature of stigma studied. The studies either (1) used existing definitions of the specific type of stigma studied, viz. enacted and felt stigma (perceived, internalized, and anticipated) (*n* = 11), (2) used existing generic stigma definitions without specifying the type of stigma (*n* = 4), (3) developed their own stigma definitions based on the disease condition studied (*n* = 4), or (4) did not explicitly define the type of stigma studied (*n* = 7). Six studies specifically termed and explored disease-specific stigma, viz. diabetes-related stigma, lung cancer-related stigma, and felt and enacted stigma as a result of disfigurement from head and neck cancer [9,10,27,28,58,59]. However, only the studies on lung cancer-related stigma [10,59], type 1 diabetes [27], and head/neck cancer-related disfigurement [58] provided their own self-developed definition of stigma describing the specific stigmatizing characteristics of the conditions. Table 2 shows the different definitions of stigma used in the studies.

### 3.2. Stigma Prevalence/Level, Tools, and Observed Correlates

The quantitative studies mostly assessed and measured three stigma types: perceived stigma (*n* = 8), experienced/enacted stigma (*n* = 3), and internalized stigma (*n* = 2). Five studies reported on the prevalence of perceived stigma ranging from 12% to 86% [12,37,60,61,62] and three on experienced stigma ranging from 5.6% to 68.5% [30,60,61]. Six studies measured and reported the mean levels/scores of stigma, the range of which varied based on the scale used [10,12,38,59,63,64]. Table 3 shows the reported prevalence and/or mean levels/scores of stigma across different NCDs.

The most common stigma measures used were the Cataldo Lung Cancer Stigma Scale (CLCSS), used by two studies [10,59] to assess perceived lung cancer stigma, and the Social Impact Scale (SIS) by two studies—one of which used it to assess perceived stigma related to lung cancer [63], while the other used it to assess internalized stigma related to prostate cancer [64]. Meanwhile, four studies developed their own questionnaire/scale/index to assess stigma [30,60,61,62], and seven studies used existing stigma scales [10,12,37,38,59,63,64]. Eight studies reported using pre-validated tools to measure stigma [10,12,37,38,59,61,63,64], whereas three did not report on the validity of the tool used [30,60,62]. Seven studies reported acceptable internal consistency of the tools used ranging from 0.73 to 0.97 [10,37,38,59,60,63,64]. In regard to the statistical associations assessed in the quantitative studies, most were on the psychological correlates/factors, viz. depression [10,12,59,60,61,63], anxiety [10,59], self-esteem [38,61], and overall health status of participants, viz. quality of life and well-being [37,59,64]. Table 3 presents the summary of the stigma tools used, their corresponding validity and reliability, and the main correlates investigated with effect sizes.

In regard to qualitative tools, most (*n* = 14) used semi-structured interview guides, while two studies [36,65] used an unstructured interview technique in which the respondents were asked to narrate their stories without much interference from the interviewer. All interview guides were reported to have been developed through consultation and discussion among authors or other stakeholders.

### 3.3. Sources of Stigma

The sources of stigma, which extrinsically perpetuate, facilitate, and reinforce stigma, were divided into two categories—structural and societal actors (public). The structural sources that were identified in the articles were media (propagation of misinformation) [9,10,28,59], healthcare (health facilities, services, and policies) [10,31], workplace (human resources, policies, and facilities) [30,33], and societal norms (negative views/connotations associated with disability, disease, and death) [8,28,33]. These sources perpetuated the stigmatization of people living with the conditions through discriminatory policies and practices [9,10,30], and the creation and reinforcement of negative stereotyping and portrayal of those living with stigmatized conditions [8,9,60]. The other source of stigma identified in the articles included societal actors who played part in stigmatization as a result of the influence and enforcement of societal norms which discredit and devalue those with health conditions, viz. family [8,9,11,28,29,31,32,36,38,59], friends [9,28,29,31,32,38,59], doctors/healthcare providers [9,28,29,34,38,59], employers [30,33,62], and colleagues at the workplace [9,38,62]. Most studies identified and examined the role of family and friends as perpetuators of stigma, followed by healthcare providers, employers, and work colleagues.

### 3.4. Biopsychosocial Mechanisms Driving NCD-Related Stigma

Three main biopsychosocial mechanisms/drivers of NCD-related stigma were identified from the review: (1) blame from others, where people living with NCDs were believed to be responsible for their condition; (2) physical symptoms/appearance, whereby visible signs, disabilities, use of medical equipment for management of their condition, and physical limitations were responsible for stigmatization from other people; and (3) fear, where the fear of contracting the disease because of misinformation and fear related to the condition and its association with death were the reasons for stigmatization from other people. Even though many NCDs shared one or more of these three causative factors, the origin and nature of how these factors instigated stigma were different and dependent on the different disease-specific features and the negative stereotypes and perceptions associated with them.

Of the ten studies which reported blame from others as a causative factor, there were clear differences across the different conditions on the causes of blame based on prevalent stereotypes. People with COPD and lung cancer were blamed for smoking as both the conditions were believed by the public to be smoking-related [8,34,65]. In the case of both type 1 and type 2 diabetes, participants felt blame from others for their poor eating habits, being overweight, and not being able to control their condition [9,27,28,66]. In the case of type 1 diabetes, the blame was perpetuated because of the belief that type 1 diabetes is like type 2 caused by lifestyle mismanagement [27]. Studies on cancer-related stigma reported different reasons for blame for different cancer types and different contexts. Gupta et al. [32] and Nyblade et al. [11] reported that in India, people with cancer were blamed for their sins, bad deeds, or karma. Nyblade et al. noted that in the case of women with breast cancer or cervical cancer, the blame was also attributed to their sexual deviance and transgressions [11].

Physical symptoms as a causative factor of NCD stigma were reported by nine studies. Among them were: physical manifestations of COPD, which include coughing spells and physical limitations [8], physical limitations and/or disability as a result of stroke [12], noticeable asthma symptoms like cough/wheeze/breathlessness (*reported by 74% participants; n = 72*) [37], and being stigmatized as a result of visible signs from the therapy (hair loss, frailty) and body disfigurement after mastectomy [35]. Other studies on cancer also reported physical signs and their severity, and the easy recognition of the condition from the appearance/disability as a driver of stigma [10,30,60]. Cataldo et al. reported on the association between severity of lung cancer and stigma (*β: 0.140, p < 0.05*) [10], while Park et al. found that cancer patients with disability had higher chances of experiencing discrimination in the workplace (*OR: 4.82, CI: 2.07–11.20*) [30]. Equipment-related cues were reported by three studies [8,27,28]. Berger et al. [8] reported the use of inhalers or oxygen by COPD patients and people with type 1 diabetes who inject insulin were reportedly mistaken for illicit drug users. Such use of medical equipment perpetuated negative judgment from others [27,28].

Six studies reported fear as a factor driving NCD stigma. Fear was mentioned mostly in studies on cancer conditions and arose from the misunderstanding that the condition is infectious and can be transmitted to others [11,32,35]. Another reason for fear was the association of cancer with death which brought about morbid feelings and uneasiness, and also the misconception that the condition may be contagious further fueled the fear of death [11,33]. An overview of the biopsychosocial mechanisms that drive NCD-related stigma is provided in Supplementary Table S4.

### 3.5. Manifestations of NCD-Related Stigma

The manifestations of NCD-related stigma in individuals that were identified from the studies were categorized into two domains: (1) enacted stigma and (2) felt stigma (perceived, internalized, and anticipated). Among enacted stigma, the main experiences reported in the articles were blame or negative judgment from doctors/healthcare providers, relationship distancing, sympathy or over-concern, social exclusion, and workplace discrimination. People with COPD, diabetes, and lung cancer reported negative judgment from doctors by insinuating their condition was self-inflicted [8,28,34,65,66]. Meanwhile, experiences of social exclusion and distancing in relationships were reported across the conditions, and those with COPD, breast and cervical, and head and neck cancers also reported too much sympathy and over-concern from others to be stigmatizing [8,11]. Discrimination in the workplace was reported by seven articles which ranged from experiences of avoidance by colleagues and supervisors to exclusion from decision-making opportunities and denying opportunities to assume important tasks and responsibilities [9,28,30,31,33,38,60].

The felt stigma domain was categorized into perceived, internalized, and anticipated stigma. In the perceived stigma domain, the main experiences identified were perception of unfair treatment and perception of being judged by others. People across different conditions perceived being unfairly treated and judged at home, work, and in healthcare settings because of their health condition [9,11,31,33]. The two main experiences of internalized stigma were self-blame and guilt and feelings of shame for having the condition. People with COPD, type 2 diabetes, and breast and cervical cancer reportedly felt ashamed and experienced feelings of self-blame and guilt for developing the condition, usually perpetuated by blame and judgment from societal actors like family, friends, and doctors. [9,32,34]. Fear of enacted stigma was the main experience under anticipated stigma experienced by people living with all four NCDs, where they feared being discriminated against in social, work, and healthcare settings because of their diseased status [8,11,28,29,32,35,36,58]. An overview of the different experiences of NCD-related stigma is shown in Supplementary Table S4.

### 3.6. Consequences of NCD-Related Stigma

Four distinct consequences of NCD-related stigma were identified from the studies: (1) social, (2) psychological, (3) behavioral, and (4) medical. Supplementary Table S4 provides an overview of the consequences of NCD-related stigma. The main social consequences of NCD-related stigma investigated were severed relationships, social isolation, and reduced work and employment opportunities. Only one study [28] reported broken/severed personal relationship because of NCD-related stigma, while most studies reported social isolation either by self or through avoidance by others [8,9,11,27,38,62], and a negative impact on work/job ranging from reduced opportunity for growth and promotion to demotion or even expulsion from the job [8,9,11,27,38,62]. Lee et al. found that perceived diabetes stigma affected both the work (*d = 0.41, p < 0.05*) and work prospects (*d = 0.39, p < 0.05*) of people affected [62], while Park et al. did not find any association between experience of discrimination among people living with cancer (miscellaneous) and employment status or prospects of getting employed [30]. Both qualitative and quantitative studies reported psychological consequences, which included distress, depression, anxiety, and lowered self-esteem, self-worth, and patient activation/self-efficacy. The quantitative studies established associations of NCD-related stigma with depression, anxiety, and self-esteem. Four studies reported an association between perceived stigma and depression (*r = 0.562, p < 0.001; r = 0.559, p < 0.001; β = 0.19, p = 0.03; β = 0.331, p < 0.001*; respectively) [10,59,61,63], while two studies found an association between experienced/enacted stigma and depression (*OR = 2.27, p < 0.05; β = 0.156, p < 0.001*) [60,61]. Association between anxiety and perceived stigma (*r = 0.418, p < 0.001; r = 0.413, p < 0.001*) was reported by two studies [10,59], while Gredig et al. found a weak association between anxiety and both perceived (*β = 0.176, p < 0.001*) and experienced stigma (*β = −0.067, p = 0.002*) [61].

Among behavioral consequences, the main consequences were non-disclosure or concealment of the condition/disease, and impaired self-care behavior. People with diabetes type 1 and 2, and lung, breast, cervical, and miscellaneous cancers with no visible physical features of the disease reported not disclosing their conditions to others to avoid stigmatization [9,11,28,32,36]. People with asthma, type 2 diabetes, and breast cancer reported negative effects of stigma on their self-efficacy, which in turn affected their self-care behavior like adherence to required medication, diet, and exercise [29,37,38,60].

Medical consequences were the least explored with only two qualitative studies reporting low access/utilization of health services wherein people with lung, breast, and cervical cancer feared seeking healthcare services because of stigma, and two studies (one qualitative and one quantitative) reporting poor medical outcomes, where Nyblade et al. found people with breast and cervical cancer attributed stigma to worsening their condition [11], while Andrews et al. reported an association between perceived stigma and asthma control/morbidity level (*d = 0.820,p = 0.02*) [37]. 

Besides the four categories of consequences, four quantitative studies reported on the association between stigma and quality of life/well-being. While Sarfo et al. did not find any statistically significant association between perceived stigma on CVD and quality of life [12], Brown et al. and Wood et al. found associations between quality of life and perceived lung cancer stigma (*β = −0.136, p = 0.015*) [59] and internalized stigma related to prostate cancer (*β = −0.150, p < 0.001*) [64], respectively. Andrews et al. reported an association between perceived stigma on asthma and quality of life domains—physical health (*r = −0.41, p = 0.001*) and mental health (*r = −0.23, p = 0.045*) [37].

### 3.7. Mitigating Factors/Strategies for NCD-Related Stigma

Mitigating factors/strategies were grouped into two domains: (1) coping strategies (internal), and (2) social support (external). Four different coping strategies were identified from the studies—disclosure, concealment, information-seeking/improving knowledge, and positive outlook and adjustments. Of the coping strategies, studies reported disclosure as a successful strategy to overcome stigma by opening up to others and not being ashamed of one’s condition, whereas concealment was reported as a strategy used to avoid stigma [8,27,33,34,66]. Studies also reported that garnering a positive outlook and developing a positive attitude, and actively seeking information on one’s condition and improving knowledge in regard to living with the condition helped people across different conditions cope with stigma [8,28,29,31]. Social support ranged from tangible support in the form of financial assistance and services (healthcare, information, help with chores) to intangible support in the form of acceptance and emotional support (empathy, affection, and care) from family, friends, peer groups/support groups, and healthcare professionals [32,35,61,63]. An overview of the mitigating factors is shown in Supplementary Table S4.

### 3.8. Quality of Evidence

Only four studies scored 0.80 or higher (three qualitative and one quantitative) and were considered to be of very good quality [9,10,33,35]. All other studies scored within the range 0.60 to 0.79 and were considered satisfactory. The quality assessment scores for each study are listed in Table 2.

## 4. Discussion

Our review attempted to explore and improve the understanding of experiences of stigma among people living with NCDs by examining and consolidating current evidence on health-related stigma associated with the four main NCDs—cancers, cardiovascular diseases, chronic respiratory diseases, and diabetes. This review confirmed that people living with NCDs, like those with communicable diseases, may also experience stigma because of their health condition, which can negatively impact their social life, health, and management of their disease. We found similarities in the experiences and consequences of stigma across the four NCDs, but the origin and nature of the causes of stigma were different depending on the disease-specific features. This review also identified three critical gaps that require specific attention from researchers. First, we found inconsistencies in defining, categorizing, and conceptualizing stigma across the studies. To address this gap, we developed a revised framework that provides an improved conceptualization and categorization of the processes and experiences of stigma related to NCDs. Second, we observed a paucity of stigma studies particularly focusing on LMICs and those on stigma related to cardiovascular diseases. Third, most studies lacked robust study designs and methodologies to offer stronger evidence on temporal relationships of stigma-related experiences, which limited a comprehensive understanding of NCD-related stigma.

The manifestations of stigma and its consequences were largely similar across the studies. This substantiated suggestions from previous studies on health-related stigma that regardless of the disease, the overall experiences of stigma and its outcomes may be the same [7,19]. Four distinct consequences of stigma were identified from the review: social, psychological, behavioral, and medical consequences. Not many studies explored and reported medical consequences, which included lower access and utilization of health services, and poor medical outcomes. However, the majority of studies identified psychological consequences like depression and lowered self-esteem and self-efficacy, social consequences like social isolation, and behavioral consequences like non-disclosure or concealment of one’s condition and impaired self-care behavior. These are all known to ultimately affect people’s utilization of health services, and clinical outcomes of their condition [22,67,68,69,70]. Considering such consequences of stigma identified in this review can severely impact the treatment and management of NCDs [71,72], it is imperative for health policy makers and program managers to focus on reducing NCD-related stigma as a measure to effectively respond to NCDs.

We found notable differences in the origin and nature of the causes of stigma across the four NCDs. We identified three main causes of stigma in the articles, viz. visibility of physical symptoms or outward appearance (biological), and fear and blame from others (psychological) driven by the negative stereotypes prevalent in society (social). Even though many NCDs shared one or more of these three causative factors, the origin and nature of how these factors instigated stigma were different and dependent on the different disease-specific features. For example, blame was identified as a driver of stigma across the different conditions; however, the origin of blame was different for each condition. People with diabetes were blamed for their poor lifestyle choices and held accountable for poor self-management of their condition, while those with COPD and lung cancers were blamed for smoking, and those with breast and cervical cancer were often accused of sexually deviant behaviors [8,11,65,66]. This finding is consistent with the work of Rao et al. [19], which noted that while the experiences of stigma may be the same in cases of all health-related stigma, the concept, origin, and nature of stigmatization may be different across different health conditions. We adapted Engel’s [52] biopsychosocial model into the revised framework to conceptualize the causes of NCD-related stigma. The model provides an improved explanation of the existence and interplay of biological, psychological, and social factors, and how they interact and work together to drive stigma. For example, in this review, we found that patients with lung cancer and COPD with visible physical symptoms were blamed for their smoking behavior for bringing the condition onto themselves regardless of whether they were smokers or not, stemming primarily from how society dictates the association of lung cancer and COPD with smoking [8,34,65].

This review also found the duality of roles of societal actors, viz. family, friends, and healthcare professionals, and structural elements like media, in both the perpetuation and mitigation of stigma. A majority of the studies in this review identified the role of family, friends, and healthcare professionals in perpetuating stigma. However, the review also found that they can play a very important part in providing social support to those affected, thereby helping in the mitigation of stigma. This ambivalent and dual nature of family, friends, and healthcare professionals has been noted by previous studies on HIV [73,74,75,76], depression [77], and leprosy [78,79] that have largely attributed it to the individual’s relevant knowledge, familiarity, and experience in regard to the disease—or lack thereof [73,74,75,77]. Similarly, we also identified the role of media as a “double-edged sword” as it often played a part as a source of stigma through negative portrayal and stereotyping of those affected [9,59,65], but sometimes also as a supportive agency in combating health-related stigma in society by raising awareness [80,81]. It is important for researchers and health program managers to be aware and cautious of this dual nature of such structural and social elements that may act as both a source and mitigator of stigma in different contexts.

### 4.1. Research Gaps and Suggestions for the Future

Many of the studies had discrepancies in properly defining and categorizing the type of stigma with only less than half of the studies providing precise definitions, while other studies either did not specify any definition, developed their own disease-specific ones, or provided a generic definition like “a mark of discredit” drawn from the works of Goffman [13] and other notable stigma researchers [15,16,67]. This may have stemmed from a lack of proper conceptualization of the processes and experiences of stigma. Previous studies have pointed out this specific shortcoming among existing works on stigma, where researchers have struggled to properly define the type and nature of stigma being studied and provide a clear conceptual framework for their study [14,18]. Further, this may also be because stigma associated with NCDs is not as well studied and explored as infectious diseases stigma and still lacks a proper conceptual framework for a better understanding of the processes and experiences of stigma associated with the conditions. The analytical framework that we developed by revising Schabert et al.’s model for diabetes-related stigma [51] can help fill this gap by providing a greater clarity to the understanding of stigma associated with NCDs and help guide future studies focusing on the different components of NCD-related stigma.

This study indicated a noteworthy discrepancy in the coverage of different conditions. More than half of the studies in this review were primarily focused on cancers, followed by diabetes, possibly indicating not only the higher stigmatization of persons living with these conditions, but also the wider recognition in academia and society [11,32]. In contrast, there was a paucity of studies on stigma related to chronic respiratory diseases and cardiovascular diseases. Especially in the case of cardiovascular diseases, we only found one study on stroke-related stigma. There is a clear need to further explore these less studied areas to gain insights into the processes and experiences of stigma pertaining to these specific conditions. Further, we found a dearth of studies conducted in LMICs where the burden and impact of stigma may be different compared to those from developed regions. Studies have found that stigma poses a greater hindrance to accessing health services in LMICs than elsewhere [78,82]. Studies have also found that stigma resides in the social context [67,83] and may be experienced and explained differently by people with different conditions in different social contexts and its impact on people’s lives may differ accordingly [15,84]. There is, therefore, a need to carry out more studies on NCD-related stigma in general, and in LMICs in particular, where WHO has estimated that by 2020, NCDs will cause 7 out of every 10 deaths [6].

We also noted that most studies in this review scored average on the quality assessment, with only four studies with higher scores. This may be because NCD-related stigma is a relatively new area of health research undergoing further development in methods and research design warranted by newly emerging evidence and knowledge gaps. The impetus for research in this area seems to have gained momentum after the United Nations General Assembly (UNGA) convened a one of a kind high-level meeting on NCDs in 2011 to take action against the growing epidemic of NCDs and save millions of lives of those affected [39]. This is apparent from our review where we found most of the selected studies were published within the last 10 years, with over one-third of studies published just in the last two years. Further, the newer studies published in the last five years also had higher-quality scores, indicating improvements in stigma research methodologies in recent years. Overall, there is a need for stigma studies on NCDs with a robust study design and rigorous methodology to generate stronger evidence.

### 4.2. Limitations

There are certain limitations to this study that need to be considered. This review was a mixed-methods review that included studies based on diverse methods (quantitative, qualitative, and mixed-methods studies). While mixed-methods reviews can be helpful in assimilating varied evidence that can be of practical use to healthcare professionals and policy makers, researchers have often argued that they can sometimes be too broad [85]. A similar limitation was encountered in this review, where the studies were hard to compare, because of the different types of stigma explored, methodologies used, and different health conditions with different specific features involved. This limited our ability to take into account and compare the levels or severity of stigma and its determinants like duration of living with the disease. However, the broader scope of exploration and diverse forms of evidence were essential in supporting the broader aim of the review and developing the revised framework for conceptualization of NCD-related stigma. We found only cross-sectional studies that explored stigma in this review. This has limited our understanding of the temporal relationships of stigma-related experiences. Besides limitations in determining causality, possible bi-directional associations between and within the causes, experiences, and consequences of stigma could not be established. Owing to the cross-sectional design used, the studies could not also see changes in the experiences of stigma overtime. Finally, in order to limit the already broad scope of this review, we did not take into account two aspects of health-related stigma. First, our review did not assess the cultural differences that may exist across different study areas and settings. Studies have shown that stigma is contextual and can differ according to geographical location and culture [21,86]. While this review highlighted the role of social norms and contextually prevalent stereotypes in stigmatization, the specific differences originating from varied cultures were not included in the review. Second, we did not take into account the existence of multiple layers of stigma arising from other social inequities (e.g., gender, race, sexuality, etc.) that usually intersect with health-related stigma and affect stigma experiences in persons affected. While none of the reviewed studies on the four NCDs specifically addressed this concept of intersectionality, the topic has recently gained interest and traction with newer studies on NCDs like diabetes [87], sickle cell disease [43], stroke [88], and mental illnesses [89,90], featuring this important aspect of health-related stigma.

### 4.3. Implications

This review provides evidence on NCD-related stigma that may be helpful for researchers, policy makers, health practitioners, and people living with NCDs. Although other models and frameworks on health-related stigma exist [15,16,21], the revised framework developed in this review is the first to specifically focus on NCD-related stigma based on systematic assimilation of empirically grounded evidence and provides a comprehensive overview of the mechanisms, manifestations, and consequences of stigma specifically pertaining to the four main NCDs. The revised framework and the identified research gaps can help guide future researchers in the exploration for better evidence on stigma and its response. The findings also provide key information to policy makers and program managers on the causes of stigma and relevant mitigating strategies to effectively target NCD-related stigma in the community and health facilities. Policies and programs can be introduced to curb discrimination based on a person’s chronic health condition, and enhance inclusivity in different sectors of the society, e.g., zero discrimination policies in health facilities, employment, mass media, education, etc.; inclusive employment policies and practices; positive depiction/portrayal of people living with NCDs in the media, including health promotional campaigns; and interventions to foster the social participation of people affected, etc. The review provides healthcare providers with a clear indication of their impact on their patients’ experiences of stigma and the various consequences as a result of it. The healthcare providers and public health experts can use this information to foster better communication and rapport with the patients to reduce stigma and improve treatment outcomes. Most importantly, this review highlights the experiences of stigma among people living with NCDs, which may be helpful in not only advancing general awareness and understanding on NCD-related stigma and the plight of people affected by it, but also in conveying the need and urgency to address this complex problem.

## 5. Conclusions

NCD-related stigma is still an emerging area of health research with inconsistencies in defining, categorizing, and conceptualizing the processes and experiences of stigma. The revised framework developed through this review can help guide future studies in improving the understanding and conceptualization of stigma related to NCDs.

## Figures and Tables

**Table 1:** Types of stigma explored in the studies.

Type of Stigma Studied		*n*	Study
Stigma type specific	Enacted stigma	9	Bonanno et al. (2012); Browne et al. (2013); Chappel et al. (2004); Cho et al. (2013); Gredig et al. (2017); Nyblade et al. (2017); Park et al. (2010); Sarfo et al. (2017); Dyer et al. (2010)
	Felt stigma: Perceived	11	Andrews et al. (2013); Berger et al. (2011); Brown et al. (2014); Browne et al. (2013); Browne et al. (2014); Cataldo et al. (2013); Dyer et al. (2010); Gonzalez et al. (2012); Gredig et al. (2017); Gupta et al. (2015); Lee et al. (2015)
	Felt stigma: Internalized	8	Bonanno et al. (2012); Chappel et al. (2004); Gupta et al. (2015); Kato et al. (2016); Kato et al. (2017); Meacham et al. (2016); Sarfo et al. (2017); Wood et al. (2017)
	Felt stigma: Anticipated	4	Bonanno et al. (2012); Chappel et al. (2004); Sarfo et al. (2017); Nyblade et al. (2017)
Stigma type unspecific	Not specified/ generic stigma	4	Halding et al. (2011); Stergiou-Kita et al. (2016); Suwakhong et al. (2016); Trusson et al. (2017)

**Table 2:** Definitions of stigma used in the studies.

Study	*n*	Quality Score	Type of Study	Type of Stigma Studied	Definition of Stated Stigma in the Study
CRD—COPD					
Berger et al. (2011)	18	0.60	Qualitative	Perceived stigma	* Not explicitly stated.
Halding et al. (2011)	18	0.65	Qualitative	Self-blame and stigmatization	** “Stigmatization is a lack of social acceptance by broader society because of being different. Stigma is socially constructed, imposing attitudes on all members in a society. A mark of discredit is extremely powerful when it communicates highly valued beliefs in a particular society.”
CRD—Asthma					
Andrews et al. (2013)	72	0.63	Quantitative	Perceived stigma	* Not explicitly stated.
CVD—Stroke					
Sarfo et al. (2017)	200	0.63	Quantitative	Enacted stigma and felt stigma (personal stigma; family stigma; community/social stigma)	*** “Enacted stigma represents discrimination against the stigmatized person that is imposed by others; internalized or felt stigma is the fear of “enacted” stigma experienced by the stigmatized person; others—not stated.”
Diabetes Type 1					
Browne et al. (2014)	27	0.65	Qualitative	Perception of diabetes-related stigma	* Not explicitly stated.
Nishio et al. (2017)	24	0.70	Qualitative	Stigma (diabetes-specific)	**** “Negative impression and sense of shame for being a diabetes patient due to negative responses of others and their own sense of values.”
Diabetes Type 2					
Browne et al. (2013)	26	0.80	Qualitative	Perceived and experienced diabetes-related stigma	* Not explicitly stated.
Gredig et al. (2017)	3347	0.63	Quantitative	Perceived and experienced	*** “Perceived stigma means the perception of stereotypes linked with labeling. Experienced stigma—also referred to in the literature as enacted stigma—means the discrimination and exclusion experienced by those affected.”
Kato et al. (2017)	209	0.67	Quantitative	Internalized stigma	*** “Internalization of society’s negative perceptions towards an illness by someone who has that particular illness.”
Kato et al. (2016)	26	0.60	Qualitative	Internalized stigma	*** “Internalized stigma refers to the negative attitudes individuals hold toward themselves on account of their condition and/or the negative reactions of others.”
Lee et al. (2015)	125	0.63	Quantitative	Perceived stigma	* Not explicitly stated.
	**Cancer—Miscellaneous**				
Cho et al. (2013)	466	0.63	Quantitative	Enacted stigma (social discrimination)	*** “Disqualification of individuals and groups who have particular health problems.”
Gupta et al. (2015)	39	0.60	Qualitative	Perceived and internalized stigma	*** “Perceived stigma refers to the shame associated with having a condition and to the fear of being discriminated and denied basic welfare rights leading to social exclusion. Actual stigma refers to obvious discrimination, which may lead to feelings of guilt, shame and threatens one’s own identity.”
Park et al. (2010)	748	0.67	Quantitative	Enacted stigma (work-related discrimination)	*** “Discrimination was defined as the experience of any of the following: reduction in salary, denial of a promotion, loss of an opportunity to demonstrate one’s ability, change of an assignment without the employee’s agreement, being treated as if one no longer has the ability to work, difficulty in revealing the cancer diagnosis to one’s employer, or pressure from the employer to quit, retire, or change jobs.”
Stergiou-Kita et al. (2016)	40	0.80	Qualitative	Stigma (unspecific)	** “Stigma is defined as both a process and an attribute. The process of stigmatization has been described by Link and Phelan as involving three key actions. First, specific human differences are labeled as negative. Second, negative differences are linked to negative social stereotypes, and third such stereotypes are used to distinguish stigmatized individuals as “bothers” within society, resulting in separation, status loss, and discrimination. As an attribute, stigma has been characterized as a label or mark that is placed on individuals (or a group), who deviate from the norm or do not comply with established behavior rules.”
Cancer—Lung					
Brown et al. (2014)	149	0.71	Quantitative	Lung cancer stigma/perceived health-related stigma	**** “Lung cancer stigma (LCS) is a perceived health-related stigma that results from negative perceptions about the causal relationship between smoking and lung cancer.”
Cataldo et al. (2013)	144	0.83	Quantitative	Lung cancer stigma/perceived health-related stigma	**** “LCS is a perceived stigma and refers to the anticipation or fear of discrimination and an awareness of negative attitudes and actions related to lung cancer. It is a perceived HRS that is defined as a personal experience characterized by exclusion, rejection, blame, or devaluation that results from anticipation of an adverse judgment related to lung cancer.”
Chappel et al. (2004)	45	0.60	Qualitative	Felt and enacted stigma	*** “Stigma occurs when society labels someone as tainted, less desirable, or handicapped. This negative evaluation may be “felt” or “enacted.”—a felt negative evaluation refers to the shame associated with having a condition and to the fear of being discriminated against on the grounds of imputed inferiority or social unacceptability—an enacted negative evaluation refers to actual discrimination of this kind.”
Gonzalez et al. (2012)	95	0.67	Quantitative	Perceived stigma	* Not explicitly stated.
Cancer—Breast					
Meacham et al. (2016)	20	0.60	Qualitative	Internalized stigma	*** “Internalized stigma or self-stigma, is when a person with a stigmatized disease applies the negative public stigma associated with the disease to his/herself.”
Suwakhong et al. (2016)	20	0.80	Qualitative	Stigma (unspecific)	** “Stigma is constructed in order to give reasons for the stigmatized person’s inferiority and to justify perceptions of the stigmatized as a threat or to be feared by others. Stigma is a mark or sign of disgrace usually eliciting negative attitudes toward the stigmatized.”
Trusson et al. (2017)	24	0.65	Qualitative	Stigma (unspecific)	** “Stigma is the situation of the individual who is disqualified from full social acceptance.”
	**Cancer—Breast and Cervical**				
Nyblade et al. (2017)	59	0.70	Qualitative	Anticipated stigma and experienced (enacted stigma)	*** “Anticipated: fear of stigma, whether or not it is actually experienced; experienced: stigma that is enacted through interpersonal acts of discrimination.”
**Cancer-Cervical**					
Dyer et al. (2010)	19	0.60	Qualitative	Perceived and experienced stigma	* Not explicitly stated.
Cancer—Prostate					
Wood et al. (2017)	85	0.63	Quantitative	Social and internalized stigma	*** “Social stigma is the most common form of experienced and researched stigma, and it exists when the larger society expresses a sense of “otherness” toward individuals due to specific characteristics (e.g., physical deformities). Internalized social stigma, wherein the opinions and views expressed in social stigma are taken in by the stigmatized and become part of their self-concept.”
Cancer—Head and Neck					
Bonanno et al. (2012)	19	0.70	Qualitative	Felt stigma and enacted stigma (disease-specific)	**** “Felt stigma indicates the patients’ own shame about her/his disfigurement and the fear of actions of discrimination against her/him. It further indicates the fact that interaction was bothersome to patients; enacted stigma refers to episodes of discrimination against patients related to the disfigurement.”

* definitions not explicitly stated; ** use of existing generic definition of stigma; *** use of existing definition of specific stigma type; **** use of self-developed disease-specific definition.

**Table 3:** Prevalence and/or levels, measurement tools, and correlates of NCD-related stigma.

Study	Stigma Outcome/ Dependent Variable	*n* (male %)	Measurement Tool	Score Range	Validity	Reliability	Prevalence (%)/ Level of Stigma (Mean, SD)	Explored Associations with Correlates
CRD—Asthma								
Andrews et al. (2013)	Perceived stigma	72 (26.3% male)	19-item stigma scale for mental health	0 to 100 (%)	Pre-validated: details NR	α = 0.89	Perceived stigma: 86% *(51% low stigma; 21% medium stigma; 14% high stigma; 14% no stigma)*	*Asthma control/morbidity (d = 0.820, p = 0.02)* *Physical health score (r = −0.41, p = 0.001)* *Mental health score (r = −0.23, p = 0.045)*
CVD—Stroke								
Sarfo et al. (2017)	Perceived stigma *(personal stigma;* *family stigma;* *community/social stigma)*	200 (52.5%)	8-item Stigma Scale for Chronic Illness (SSCI-8)	8 to 40	Pre-validated: details NR	NR	Perceived personal stigma: 79% (13.7, SD 5.7) Perceived family stigma: 63% (11.9, SD 4.6) Perceived community stigma: 62% (11.4, SD 4.4)	Perceived personal stigma vs: *No family history of stroke (β = 1.58; p = 0.03)* *Depression (NS)* *Employment (Employed* vs. *Unemployed) (NS)* *Stroke severity (NS)* *HRQoL (NS)*
**Diabetes Type 2**								
Gredig et al. (2017)	Perceived stigma and experienced stigma	3347 (54.8% male)	33-item Experienced Stigma and 26-item Perceived Stigma (self-developed questionnaire)	0 to 100 (%)	Pre-validated: construct validity	NR	Experienced stigma: 68.5% Perceived stigma: 84.4%	Experienced stigma vs: *Self-esteem (β = −0.067; p = 0.002)* *Psychological distress (β = 0.166; p < 0.001)* *Depressive symptoms (β = 0.156; p < 0.001)* *Perceived social support (β = −0.073; p < 0.002)* Perceived stigma vs: *Self-esteem (β = −0.176; p < 0.001)* *Psychological distress (β = 0.367; p < 0.001)* *Depressive symptoms (β = 0.331; p < 0.001)* *Perceived social support (β = −0.220; p < 0.001)*
Kato et al. (2017)	Self (internalized)-stigma	209 (80.4% male)	39-item self-stigma scale in Japanese (SSSJ)	0 to 117	Pre-validated: details NR	α = 0.96	Mean self/internalized stigma: 72.5, SD 6.38	*Self-esteem and social participation (d = 0.79, p < 0.001)*
Lee et al. (2015)	Perceived stigma	125 (68% male)	8-item questionnaire to assess perceived stigma (self-developed questionnaire)	0 to 100%	NR	NR	Perceived stigma: 12%	*Diabetes affects work (d = 0.41; p < 0.05)* *Diabetes affects work prospect (d = 0.39; p < 0.05)*
**Cancer—Miscellaneous**								
Cho et al. (2013)	Perceived cancer stigma *(attitudes towards cancer)* and experienced ancer stigma *(social discrimination)*	466 (46.1% male)	12-item questionnaire to assess cancer stigma *(8-item attitudes towards cancer, and 4-items social discrimination)* (self-developed questionnaire)	0 to 100%	NR	Attitudes towards Cancer: *impossibility of recovery (α = 0.75) and stereotypes (α = 0.76)* Social discrimination (α = 0.89)	Perceived and experienced cancer stigma: 30% *[Impossibility of recovery: cancer impossible to be treated (30.8%), difficult to be healthy again (40%), cannot be socially active because of cancer (36.2%)]* *[Stereotypes: cancer patients recognized by appearance (31.3%), have difficult time having sexual intimacy (31.3%)]* *[Social discrimination: avoided by friends (10.1%), avoided by neighbors (8.2%), problems with family (10.5%), discrimination from employers and coworkers (5%)]*	Negative attitude towards cancer (Ref: positive attitude) vs: *Depression (OR: 2.72, p < 0.05)* Experience of discrimination (Ref: No) vs: *Depression (OR: 2.27, p < 0.05)*
Park et al. (2010)	Experienced stigma *(discrimination)*	748 (59.0% male)	Single-item indicator for experienced stigma and 10-items for types of discrimination experienced (self-developed questionnaire)	0 to 100%	NR	NR	Experienced discrimination in the workplace: 5.6% [*reduction in salary (27.1%), loss of opportunity to display ability (13.6%), pressure to quit or change job (13.6%), difficulty of revealing cancer diagnosis (11.9%)]*	Experienced discrimination vs: *Disability (OR: 4.82, CI: 2.07–11.20) Change in employment status (NS) Unemployed (NS)*
Cancer—Lung								
Brown et al. (2014)	Perceived health-related stigma *(lung cancer stigma)*	149 (25% male)	31-item Cataldo Lung Cancer Stigma Scale (CLCSS)	31 to 124	Pre-validated: construct validity	α = 0.96	Mean perceived stigma level: 75.9, SD 18.20	*QoL (β = −0.136, p = 0.015)* *Anxiety (r = 0.418, p < 0.001)* *Depression (r = 0.562, p < 0.001),*
Cataldo et al. (2013)	Perceived health-related stigma (lung cancer stigma)	144 (26% male)	31-item Cataldo Lung Cancer Stigma Scale (CLCSS)	31 to 124	Pre-validated: construct validity	α = 0.97	Mean perceived stigma level: 75.7, SD 18.30	*Symptom severity (β = 0.140, p < 0.05)* *Anxiety (r = 0.413; p < 0.001)* *Depression (r = 0.559, p < 0.001)* *Symptom severity (r = 0.483, p < 0.001)*
Gonzalez et al. (2012)	Perceived stigma	95 (41.1% male)	24-item Social Impact Scale (SIS)	Total score SIS (0 to 80)	Pre-validated: details NR	α = 0.81	Mean total SIS score (perceived stigma): 42.9, SD 11.87	*Depression (β = 0.19, p = 0.03)*
**Cancer—Prostate**								
Wood et al. (2017)	Self/internalized stigma and social stigma	85 (all male)	24-item Social Impact Scale (SIS): *internalized shame/stigma, social rejection, financial insecurity, social isolation*	Internalized shame/stigma (5 to13) Social rejection (9 to 20) Social isolation (7 to 23) Financial insecurity (3 to 12)	Pre-validated: details NR	Internalized shame (α = 0.73), social rejection (α = 0.84), financial insecurity (α = 0.85), social isolation (α = 0.92)	SIS internalized shame: 7, SD 2.42 SIS social rejection: 10.63, SD 2.71 SIS social isolation: 9.61, SD 3.80 SIS financial insecurity: 4, SD 1.48	SIS internalized shame vs. *QoL (β = −0.15, p < 0.001)* SIS social rejection vs. *QoL (β = 0.18, p < 0.001)* SIS social isolation vs. *QoL (β = −0.36, p < 0.001)* SIS financial insecurity vs. *QoL (β = −0.34, p < 0.001)*

NR—not reported; NS—non-significant association; α—Cronbach’s alpha; QoL—quality of life.

## Supplementary Materials

The following are available online at https://www.mdpi.com/1660-4601/17/18/6657/s1, Table S1: PRISMA checklist, Table S2: Search strategy, Table S3: Quality assessment matrix, Table S4: Integrative matrix with summary of qualitative and quantitative findings.
