# Supplementary material for: Non-Communicable Diseases-Related Stigma: A Mixed-Methods Systematic Review
Source: Int J Environ Res Public Health. 2020 Sep 12;17(18):6657. doi: 10.3390/ijerph17186657 (PMC7559120; doi:10.3390/ijerph17186657)
Supplement: Supplementary file 1 [file ijerph-17-06657-s001.zip › Table S2 Search Strategy.docx]

| **Database** | **Search strategy** | **Results** |
| --- | --- | --- |
| PubMed | ((((stigma[Title] OR discrimination[Title])) AND (cancer[Title] OR non communicable disease[Title] OR non-infectious disease[Title] OR diabetes [Title] OR stroke [Title] OR heart disease[Title] OR cardiovascular[Title] OR chronic respiratory disease[Title] OR asthma[Title] OR Obstructive Pulmonary[Title] OR angina[Title] OR ischemia[Title])) AND Humans[Mesh] AND English[lang] AND medline[sb]) | 394 |
| Web of Science | (TI=(stigma OR discrimination) AND TI=(NCD OR non communicable disease OR non infectious disease OR cancer OR diabetes OR stroke OR asthma OR cardiovascular OR chronic respiratory OR obstructive pulmonary OR CVD OR COPD OR embolism OR ischemi*)) | 439 |
| JSTOR | (((ti:(stigma OR discrimination) OR tb:(stigma OR discrimination)) AND (ti:((stroke OR heart) (disease OR cardiovascular OR CVD OR angina OR coronary) (artery OR ischemia OR atherosclerosis OR hypertension)) OR tb:((stroke OR heart) (disease OR cardiovascular OR CVD OR angina OR coronary) (artery OR ischemia OR atherosclerosis OR hypertension)))) AND la:(eng OR en) | 11 |
| PsycINFO | ti(stigma OR discrimination) AND ti(cancer OR diabetes OR stroke OR coronary artery OR asthma OR COPD OR chronic obstructive OR cardiovascular OR heart disease OR CVD OR chronic respiratory) | 32 |
| Science Direct | TITLE(stigma OR discrimination) and TITLE((chronic obstructive pulmonary OR COPD OR respiratory OR CRD OR asthma) OR (cancer) OR (diabetes) OR (stroke OR CVD OR cardiovascular)) | 135 |
| **TOTAL** | | 1011 |
